# Risk of diabetes among school going adolescents in India

**DOI:** 10.6026/973206300222249

**Published:** 2026-04-30

**Authors:** Rohit Kumar Das, Likhar S.K, Piyusha Mahashabde

**Affiliations:** 1 Department of Community Medicine, Sunderlal Patwa Government Medical College, Mandsaur, Madhya Pradesh, India; 2 Department of Community Medicine, Dr. L.N.P. Government Medical College, Ratlam, Madhya Pradesh, India

**Keywords:** Adolescents, diabetes risk, Indian diabetes risk score (IDRS)

## Abstract

Diabetes mellitus, a leading non-communicable disease (NCD), is increasingly linked to early-life behaviors and risk factors established during adolescence. Early detection among youth is crucial for prevention. Therefore, it is of interest to assess the risk of diabetes and its associated factors among school-going adolescents aged 15-19 years in Ratlam, Madhya Pradesh. Data were collected using a pre-tested questionnaire covering socio-demographic profile, lifestyle behaviors. Anthropometric measurements and blood pressure were recorded following WHO protocols. Diabetes risk was assessed using the Indian Diabetes Risk Score (IDRS) and statistical analysis was performed using chi-square tests and logistic regression. Most participants (91.3%) were categorized as low risk for diabetes, while 8.4% were at moderate risk and 0.3% at high risk. Elevated Body Mass Index (BMI) (Adjusted OR: 4.116; p = 0.002) and elevated blood pressure (Adjusted OR: 2.950; p = 0.020) emerged as significant predictors of diabetes risk. Waist circumference was also significantly higher among adolescents with moderate/high IDRS scores (p = 0.002).

## Background:

Non-communicable diseases (NCDs) have emerged as a major global health concern, contributing significantly to morbidity and mortality, particularly in adulthood [1]. However, evidence increasingly suggests that the origins of these diseases are often rooted in early life stages, including childhood, adolescence and even in utero [2]. Symptoms are usually rapid in onset and include polyuria, polydipsia, nausea, abdominal pain, vomiting [3]. Lifestyle behaviors adopted during childhood and adolescence-especially those related to diet, physical activity and sedentary habits have a profound and lasting impact on long-term health outcomes. Unhealthy behaviors initiated during these developmental periods and sustained into adulthood account for approximately 70% of premature adult deaths [4]. Numerous studies have demonstrated that such early life risk behaviors significantly increase the likelihood of developing chronic conditions such as cardiovascular disease, obesity, type 2 diabetes, hypertension and certain cancers [5]. In rural areas, awareness and preventive action remain limited. The findings underscore the urgent need for targeted preventive strategies and health education initiatives aimed at reducing the burden of NCDs among youth, particularly in underserved regions [6, 7, 8-9]. Therefore, it is of interest to explore the risk of diabetes among adolescents, assess their awareness levels and evaluate the impact of lifestyle behaviors on early health outcomes.

## Methodology:

This community-based cross-sectional study was conducted in Ratlam city, Madhya Pradesh, from September 2022 to March 2024. The study aimed to assess the risk factors and level of awareness regarding diabetes among school-going adolescents aged 15-19 years. It was carried out under the Department of Community Medicine, Government Medical College, Ratlam and included both government and private schools within the city. The study population comprised adolescents enrolled in secondary and higher secondary schools. A total of 334 participants were selected using a simple random sampling method. The sample size was calculated based on the NFHS-5 reported prevalence of overweight and obesity among males aged 15 years and above (25.5%). Using the standard formula (N = 4PQ/D^2^) with a 5% margin of error and 95% confidence level, the calculated sample size was 304. After accounting for a 10% non-response rate, the final sample size was adjusted to 334. Schools were selected at random from a list provided by the District Education Officer (DEO) and within each selected school, students who met the inclusion criteria (aged 15-19 years and present on the day of data collection) were also selected randomly. Students who were absent or unwilling to participate were excluded. Ethical clearance was obtained from the Institutional Ethical Committee of Government Medical College, Ratlam. Written permissions were secured from the DEO and school principals. Informed consent and assent were obtained from all participants prior to data collection, ensuring confidentiality and voluntary participation. Data were collected using a pre- designed, pre-tested, semi-structured questionnaire. It included sections on sociodemographic characteristics, dietary habits, physical activity (assessed using WHO's Global Physical Activity Questionnaire - GPAQ), family history of diabetes and knowledge and awareness of diabetes. Anthropometric measurements such as height, weight, waist and hip circumference were recorded following WHO protocols. Blood pressure was measured using a digital sphygmomanometer. The risk of diabetes was assessed using the Indian Diabetes Risk Score (IDRS), based on factors such as age, waist circumference, physical activity and family history. Participants were categorized into low (<30), moderate (30-50) and high risk (>60) groups. Data were entered into Microsoft Excel and analyzed using the free version of SPSS. Statistical analysis included descriptive statistics, chi-square tests, independent t-tests and logistic regression models to identify significant associations and predictors of high diabetes risk among adolescents.

## Results:

Out of the total 334 school-going adolescents aged 15-19 years who participated in the study, the majority 91.3% (n = 305) were classified as having a low risk for diabetes according to the Indian Diabetes Risk Score (IDRS). However, 8.4% (n = 28) of the participants were in the moderate-risk category and 0.3% (n = 1) was categorized as having a high risk (IDRS > 60). This indicates that nearly one in ten adolescents may already be at a moderate or high risk of developing diabetes (Table 1 - see PDF). When disaggregated by gender, a slightly higher proportion of girls (9.3%) were found to be at moderate or high risk compared to boys (7.9%). In univariate analysis, several potential risk factors were examined for their association with elevated diabetes risk (IDRS > 30). Among these, elevated Body Mass Index (BMI) showed a strong and statistically significant association with increased diabetes risk (OR: 5.65, 95% CI: 2.38-13.4; p < 0.001). Similarly, elevated blood pressure (hypertension) was also significantly associated with higher IDRS scores (OR: 3.85, 95% CI: 1.60-9.29; p = 0.001). Other variables such as gender, type of school, diet type, fruit and fast-food consumption were not significantly associated with increased risk in univariate analysis, though some trends were noted (Table 2 - see PDF). Bivariate analysis further revealed that participants in the moderate/high-risk IDRS group had a significantly higher mean waist circumference compared to those in the low-risk group (77.0 cm vs 72.3 cm, p = 0.002) However, waist-hip ratio did not differ significantly between groups. Multivariate logistic regression confirmed that both elevated BMI (Adjusted OR: 4.116, 95% CI: 1.678-10.095; p = 0.002) and elevated blood pressure (Adjusted OR: 2.950, 95% CI: 1.184-7.350; p = 0.020) were independent predictors of increased diabetes risk among adolescents. These findings suggest an emerging burden of diabetes risk even in the adolescent population and highlight the importance of early screening and intervention, particularly targeting obesity and hypertension as modifiable risk factors.

## Discussion:

The present study assessed the risk of diabetes among school-going adolescents aged 15-19 years in Ratlam, Madhya Pradesh, using the Indian Diabetes Risk Score (IDRS). The analysis revealed that the majority (91.3%) of participants fell into the low-risk category, with 8.4% at moderate risk and a small fraction (0.3%) classified as high risk. Although the proportion of high-risk individuals is low, the presence of moderate risk in nearly one in every twelve adolescents is noteworthy and indicative of emerging health concerns at an early age. These findings are consistent with other Indian studies that have examined diabetes risk in adolescents and young adults [7]. The comparatively lower prevalence observed in the current study may be due to its setting in a semi-urban area, different lifestyle factors, or greater prevalence of traditional diets and physical activity levels in this region. One of the most important findings from this study is the statistically significant association between elevated Body Mass Index (BMI) and diabetes risk. Adolescents who were overweight or obese were found to be over five times more likely to have an IDRS score above 30 in univariate analysis (OR = 5.65, p < 0.001) and the association remained significant after adjustment for other variables in multivariate logistic regression (Adjusted OR = 4.116, p = 0.002). Likewise, elevated blood pressure was also a strong predictor of increased diabetes risk (Adjusted OR = 2.950, p = 0.020), confirming findings from earlier studies which emphasized hypertension as a crucial early indicator of metabolic syndrome. Anthropometric measurements, particularly waist circumference, were also significantly higher in those with moderate to high IDRS scores (p = 0.002), further supporting the role of central obesity in diabetes pathogenesis. Interestingly, the waist-hip ratio and dietary variables such as fruit intake, junk food consumption and type of diet did not show statistically significant associations. This could be due to limitations in the self-reported nature of these variables or a lack of variation in dietary habits across the sample population. Although gender was not found to be a significant factor in diabetes risk, slightly more girls were in the moderate- or high- risk groups compared to boys. This may reflect broader gendered trends in physical activity, dietary behavior, or access to health knowledge and services and warrants further exploration in future research. Overall, the findings underscore the need for early screening and targeted health interventions among adolescents [8]. The use of a simple, cost-effective tool like the IDRS can help identify those at risk in school and community settings. Integrating such screening with school health programs, physical activity promotion and nutritional education could significantly reduce the future burden of diabetes and related non-communicable diseases.

## Conclusion:

The adolescent age group is more vulnerable to the Type 2 diabetes due to rapidly changing life style. Although the proportion of high- risk individuals is low, the presence of moderate risk in nearly one in every twelve adolescents is noteworthy and indicative of emerging health concerns at an early age. Hence, tailored lifestyle interventions are needed to tackle the problem.

## Source of Funding:

None
